# Predictors of diagnostic conversion from major depression to bipolar disorder: a Swedish national longitudinal study

**DOI:** 10.1017/S0033291723001848

**Published:** 2023-12

**Authors:** Sang Jin Rhee, Henrik Ohlsson, Jan Sundquist, Kristina Sundquist, Kenneth S. Kendler

**Affiliations:** 1 Biomedical Research Institute, Seoul National University Hospital, Seoul, Korea; 2 Center for Primary Health Care Research, Lund University, Malmö, Sweden; 3 Department of Family Medicine and Community Health and Department of Population Health Science and Policy, Icahn School of Medicine at Mount Sinai, New York, NY, USA; 4 Virginia Institute for Psychiatric and Behavioral Genetics, Virginia Commonwealth University, Richmond, VA, USA; 5 Department of Psychiatry, Virginia Commonwealth University, Richmond, VA, USA

**Keywords:** Bipolar disorder, conversion, family genetic risk scores, major depression

## Abstract

**Background:**

It is clinically important to predict the conversion of major depression (MD) to bipolar disorder (BD). Therefore, we sought to identify related conversion rates and risk factors.

**Methods:**

This cohort study included the Swedish population born from 1941 onward. Data were collected from Swedish population-based registers. Potential risk factors, including family genetic risk scores (FGRS), which were calculated based on the phenotypes of relatives in the extended family and not molecular data, and demographic/clinical characteristics from these registers were retrieved. Those with first MD registrations from 2006 were followed up until 2018. The conversion rate to BD and related risk factors were analyzed using Cox proportional hazards models. Additional analyses were performed for late converters and with stratification by sex.

**Results:**

The cumulative incidence of conversion was 5.84% [95% confidence interval (95% CI) 5.72–5.96] for 13 years. In the multivariable analysis, the strongest risk factors for conversion were high FGRS of BD [hazard ratio (HR) = 2.73, 95% CI 2.43–3.08], inpatient treatment settings (HR = 2.64, 95% CI 2.44–2.84), and psychotic depression (HR = 2.58, 95% CI 2.14–3.11). For late converters, the first registration of MD during the teenage years was a stronger risk factor when compared with the baseline model. When the interactions between risk factors and sex were significant, stratification by sex revealed that they were more predictive in females.

**Conclusions:**

Family history of BD, inpatient treatment, and psychotic symptoms were the strongest predictors of conversion from MD to BD.

Although Kraepelin (1899) proposed a unitary concept of manic-depressive insanity, the dichotomy of affective disorders into major depression (MD) and bipolar disorder (BD), initially proposed by Karl Leonard (Perris, 1990), has become the official nosology since DSM-III (American Psychiatric Association, 1980) and ICD-9 (World Health & International Conference for the Ninth Revision of the International Classification of Diseases, 1977). Studies have indeed revealed that these disorders differ in several important ways, including the nature of their genetic liability (Kendler, Ohlsson, Sundquist, & Sundquist, 2022), prognosis (Kessing, Hansen, & Andersen, 2004), and effective pharmacological treatment (Bauer, Severus, Möller, & Young, 2017; Grunze et al., 2010). However, a substantial proportion of BD patients experience their initial episode as MD (Baldessarini, Tondo, & Visioli, 2014). Therefore, in these patients, an inevitable time lag exists before the diagnosis of BD, which could take place when a manic episode first occurs (Ghaemi, Sachs, Chiou, Pandurangi, & Goodwin, 1999). Misdiagnosis can lead to suboptimal treatment. In particular, antidepressant monotherapy in BD increases the risk of (hypo)manic states and conversion to a rapid cycling course (Baldessarini et al., 2013; Mattes, 2006). Thus, there have been numerous studies that have investigated the conversion of MD to BD. We have reviewed 28 significant studies that have been published since 1999 in Table 1. Conversion risks varied from 2% to 41% (Goldberg, Harrow, & Whiteside, 2001; Kim et al., 2020a), with much of this variation likely arising from differences in study design. For example, rates of conversion likely decrease with time; therefore, different observation durations would influence the observed conversion rates (Kessing, Willer, Andersen, & Bukh, 2017).

**Table 1.** Review of previous significant risk factors of MD to BD conversion

Characteristics		Studies			Strength of risk factor^a^
		Protective factor	Not significant	Risk factor	
Early age of onset		Baryshnikov et al. (2020); James et al. (2015)	Musliner and Østergaard (2018); Bukh et al. (2016); Nakamura et al. (2015); Gilman, Dupuy, and Perlis (2012); Takeshima et al. (2008); Holma et al. (2008); Angst et al. (2005); Goldberg et al. (2001)	Xu et al. (2022); Jo et al. (2022); Tse et al. (2021); Oliveira et al. (2021); Kim et al. (2020b); Kim et al. (2020b); Woo et al. (2015); Tondo, Visioli, Preti, and Baldessarini (2014); Dudek et al. (2013); Fiedorowicz et al. (2011); Gan et al. (2011); Beesdo et al. (2009); Wong et al. (2009); Othmer et al. (2007); Kessing (1999)	++
Female sex		Kim et al. (2020b); Tondo et al. (2014); Wong et al. (2009)	Xu et al. (2022); Jo et al. (2022); Tse et al. (2021); Oliveira et al. (2021); Pfennig et al. (2016); Bukh et al. (2016); Woo et al. (2015); Gilman et al. (2012); Fiedorowicz et al. (2011); Gan et al. (2011); Beesdo et al. (2009); Takeshima et al. (2008); Holma et al. (2008); Angst et al. (2005); Goldberg et al. (2001); Kessing (1999)	Baryshnikov et al. (2020); de Azevedo Cardoso et al. (2020); Kim et al. (2020b); Musliner and Østergaard (2018); James et al. (2015)	–
Familial history	Mental disorder		Tse et al. (2021); Oliveira et al. (2021); Nakamura et al. (2015); Gan et al. (2011); Wong et al. (2009)	Tondo et al. (2014)	–
	Affective disorder		Xu et al. (2022); Holma et al. (2008)	Bukh et al. (2016); Tondo et al. (2014)	–
	BD		de Azevedo Cardoso et al. (2020); Bukh et al. (2016); Takeshima et al. (2008); Angst et al. (2005); Goldberg et al. (2001)	Oliveira et al. (2021); Musliner and Østergaard (2018); Woo et al. (2015); Tondo et al. (2014); Fiedorowicz et al. (2011); Wong et al. (2009); Othmer et al. (2007)	+ +
	MD		Gilman et al. (2012); Fiedorowicz et al. (2011)	Musliner and Østergaard (2018)	–
Depression characteristics					
Psychotic symptoms			Xu et al. (2022); de Azevedo Cardoso et al. (2020); Bukh et al. (2016); Nakamura et al. (2015); Woo et al. (2015); Wong et al. (2009); Takeshima et al. (2008); Holma et al. (2008)	Tse et al. (2021); Baryshnikov et al. (2020); Kim et al. (2020b); Musliner and Østergaard (2018); James et al. (2015); Fiedorowicz et al. (2011); Gan et al. (2011); Othmer et al. (2007); Goldberg et al. (2001)	+ +
Severity			Bukh et al. (2016)	Baryshnikov et al. (2020); Musliner and Østergaard (2018); Holma et al. (2008)	+
Multiple depressive episodes/recurrent depression			Oliveira et al. (2021); Nakamura et al. (2015); Woo et al. (2015); Gilman et al. (2012); Fiedorowicz et al. (2011); Gan et al. (2011); Holma et al. (2008)	Baryshnikov et al. (2020); Kim et al. (2020b); Musliner and Østergaard (2018); Tondo et al. (2014); Dudek et al. (2013); Takeshima et al. (2008); Angst et al. (2005)	–
Suicidal acts/self-harm		Tse et al. (2021)	Xu et al. (2022); de Azevedo Cardoso et al. (2020); Kim et al. (2020b); Pfennig et al. (2016); Bukh et al. (2016); Nakamura et al. (2015); Wong et al. (2009)	Kim et al. (2020a); Tondo et al. (2014); Gan et al. (2011)	–
Hospitalization/inpatients			de Azevedo Cardoso et al. (2020); Fiedorowicz et al. (2011); Holma et al. (2008); Wong et al. (2009)	Kim et al. (2020a, 2020b); Musliner and Østergaard (2018)	–
Comorbidity					
Substance use disorder			Kim et al. (2020b); Pfennig et al. (2016); Goldberg et al. (2001)	Kim et al. (2020a); Bukh et al. (2016); Chen et al. (2015); Tondo et al. (2014)	+
Alcohol use			Oliveira et al. (2021)^b^; de Azevedo Cardoso et al. (2020)^b^; Gilman et al. (2012); Fiedorowicz et al. (2011); Wong et al. (2009); Holma et al. (2008)	Musliner and Østergaard (2018); Chen et al. (2015)	–
Drug use			de Azevedo Cardoso et al. (2020) (other than cocaine);^b^ Musliner and Østergaard (2018) (substance use disorder excluding alcohol); Gilman et al. (2012); Fiedorowicz et al. (2011); Wong et al. (2009)	Oliveira et al. (2021)^b^; de Azevedo Cardoso et al. (2020) (cocaine)^b^; Gan et al. (2011)	–
Anxiety disorder		Kim et al. (2020b)	de Azevedo Cardoso et al. (2020); Musliner and Østergaard (2018); Pfennig et al. (2016); Bukh et al. (2016); Gilman et al. (2012) (panic disorder); Gan et al. (2011); Fiedorowicz et al. (2011) (panic disorder, phobia, GAD); Takeshima et al. (2008); Holma et al. (2008) (other than social phobia)	Kim et al. (2020a); Chen et al. (2015); Gilman et al. (2012) (social phobia, GAD); Holma et al. (2008) (social phobia)	–
OCD			Kim et al. (2020b); Musliner and Østergaard (2018); Fiedorowicz et al. (2011)	Kim et al. (2020a); Holma et al. (2008)	–
ADHD			de Azevedo Cardoso et al. (2020); Kim et al. (2020b); Musliner and Østergaard (2018); Pfennig et al. (2016); Tondo et al. (2014)	Kim et al. (2020a); Chen et al. (2015)	–
Psychotropic medication					
Antipsychotics				Kim et al. (2020b)	–
Mood stabilizer			Nakamura et al. (2015)	Kim et al. (2020b); Goldberg et al. (2001)	+
Antidepressants			Oliveira et al. (2021); Kim et al. (2020b); Goldberg et al. (2001)	Jo et al. (2022); Kim et al. (2020a)	–

MD, major depressive disorder; BD, bipolar disorder; OCD, obsessive-compulsive disorder; ADHD, attention deficit hyperactivity disorder.

Results are based on the main multivariate analysis of the study unless only a univariate analysis was performed. The results of Oliveira et al. (2021) and de Azevedo Cardoso et al. (2020) and the results of Pfennig et al. (2016) and Beesdo et al. (2009) are from the same study.

a ++: A risk factor that was replicated, if there are more significant than non-significant studies, and a significant risk factor from the pooled meta-analysis of Ratheesh et al. (2017), +: A risk factor that was replicated, and if there are more significant than non-significant studies, and –: others.

b Based on lifetime use.

We also reviewed significant risk factors in these studies and summarized them in Table 1. Early age of onset was the most consistently and frequently reported risk factor for conversion. However, the effect of sex was controversial; the majority of the studies reported it as non-significant. Familial loadings were analyzed in various ways, and the presence of BD familial history was frequently reported as a risk factor. When considering depression characteristics, those with certain severe features (psychotic features, severity, and multiple depressive episodes/recurrent depression) had more positive results, but other severity features (suicidal acts/self-harm and hospitalization/inpatients) had more negative results. Substance use disorders alone had more reports of significance but had fewer reports when these were analyzed separately as alcohol use disorder (AUD) or drug use disorder (DUD). Finally, even though the effects of medications, especially of antidepressants, have been identified as risk factors in multiple prior reports (Baldessarini et al., 2013), only a few studies compared its effect with other risk factors above and, interestingly, only mood stabilizers had more reports of significance.

Several recent meta-analyses of longitudinal studies have been performed. Ratheesh et al. (2017) excluded register-based studies and identified early age of onset of MD, the presence of psychotic symptoms, and the presence of BD family history as significant risk factors in pooled analysis. Kessing et al. (2017) included two national register-based studies (Chen et al., 2015; Kessing, 1999), and analyzed if each risk factor was consistently replicated throughout the studies it reviewed. As also seen in our review in Table 1, the meta-analysis reported that all of the risk factors that had positive results also had reports of negative results.

The aim of this national register-based study using Swedish data was to determine (1) the conversion rate across time, (2) the risk factors of conversion of patients with MD to BD, and (3) different risk factors for different conversion durations and sex. Our study was performed to overcome some of the limitations of previous longitudinal studies. First, this study was performed on national registers to enhance generalizability. Second, we utilized a relatively long follow-up period to observe conversion to BD. Third, we utilized FGRS (family genetic risk scores), which enabled us to analyze genetic liability using a quantitative index.

## Methods

We collected information on individuals from Swedish population-based registers with national coverage linking each person's unique personal identification number, which, to preserve confidentiality, was replaced with a serial number produced by Statistics Sweden. We secured ethical approval for this study from the Regional Ethical Review Board in Lund (No. 2008/409 and later amendments). Our database consisted of all individuals born in Sweden to Swedish-born parents from 1941 onward, followed up through to 31 December 2018. In the database, we included date of registration for MD, BD, anxiety disorder (AD), other nonaffective psychosis (ONAP), obsessive-compulsive disorder (OCD), DUD, AUD, suicide attempt (SA), and attention deficit hyperactivity disorder (ADHD), utilizing ICD-8, 9, and 10 codes from Swedish national Specialist and Hospital registries, almost nationwide primary care data, as well as information from Prescription and Criminal registers for DUD and AUD. From the 21 counties in Sweden, the primary care data covered 16 counties/90% of the population by 2009 and 19 counties/96% of the population by 2011 (Appendix Table 1). For MD, we also included information on the type of diagnosis (mild, moderate, severe, psychotic), site of registration (treatment setting), and medication (Appendix Table 2). The number of registrations of MD or BD was defined by the total number of registrations of all registers. Registrations within 90 days from the previous registration were assumed to represent the same episode and so were not counted. Furthermore, we included individual FGRS for MD and BD. The FGRS are calculated from morbidity risks for disorders in 1^st^–5^th^ degree relatives, controlling for cohabitation effects, sex, age, and area of residence and, thus, arise from phenotypes in extended relatives (obtained from the Multigeneration register), not from molecular data. In the models, the FGRS were divided into four groups that were defined by K-means clustering (Appendix Table 3).

For analysis, we selected all individuals with a first registration for MD during the period 2006 to 2018, who were 12 years or older at the time of registration. In addition, we required that the individuals were not registered for BD or schizophrenia prior to their registration for MD. Most registrations for MD occur in primary care. Therefore, in order to ensure that we captured the first episode of MD, we required that an individual had to reside, at least one year prior to their registration, in a county that was included in our primary care data. In total, we investigated 641 064 individuals (93% of all individuals with a MD registration in our study period).

To study conversion from MD to BD, we used Cox regression analysis where follow-up time was measured until the first registration for BD, death, emigration, or the end of follow-up (2018-12-31), whichever occurred first. First, we performed univariable analyses for all variables. In the second step, we fitted a multivariable Cox regression model using all predictors. Note that all predictors were measured at or prior to the first MD episode. We repeated the analyses by requiring at least two independent BD registrations for the onset of BD with the second registration as the endpoint, as only one BD registration might produce false positives for a ‘true’ conversion to BD. We then split the sample randomly into a training and test set (50:50). A multivariable Cox regression model was performed in the training set and was applied to the test set. Its performance in the test set was evaluated with concordance statistics and receiver operating characteristic (ROC) curves (Kamarudin, Cox, & Kolamunnage-Dona, 2017). We then created a risk score based on the deciles of the linear predictor and used it as a predictor variable in a Cox regression model. In the next step, we replicated the analyses for those individuals who did not convert during the first five years after their first MD registration. In this model, we added information on the same variables as in the initial model but now also included information on what had occurred during the five years after the initial MD registration (e.g. new registrations of psychiatric comorbidities, number of MD registrations). In this model, we also included information on treatment with medication.

Additionally, the analysis was stratified by sex to explore sex-dependent risk factors. Specifically, at first, the interaction terms with sex were analyzed in a univariate Cox regression model for each predictor. Statistical significance was considered after correction for multiple comparisons with the Bonferroni method. We then performed a multivariable Cox regression model for each sex. The final statistical significance for sex differences was based on the significance of both the univariate interaction analysis and the multivariable Cox regression.

All statistical analyses were performed using SAS 9.4.

## Results

### Descriptive analysis

As outlined in Table 2, we identified 641 064 patients with MDD registrations from 2005 to 2018, of whom 62.7% were females. The majority (78.8%) were from primary care settings, and 38.7% had comorbid AD (Table 2). During the follow-up period, a total of 20 750 patients (3.24%) converted to BD (Table 3). The cumulative incidence of conversion was 5.84% (95% CI 5.72–5.96) for 13 years, and the median follow-up duration was 5.1 years (Appendix Figure 1). The mean number of affected relatives in each FGRS group for MD and BD is presented in Appendix Table 4.

**Table 2.** Demographic and clinical characteristics

Characteristics	All patients (*N* = 641 064)
Sex	
Males	238 743 (37.3%)
Females	402 321 (62.7%)
FGRS_BD_	
Low (−0 s.d.)	530 957 (82.8%)
Mid-low (0–1 s.d.)	70 646 (11.0%)
Mid-high (1–2 s.d.)	32 224 (5.0%)
High (2 + s.d.)	7237 (1.1%)
FGRS_MD_	
Low (−0 s.d.)	180 134 (28.1%)
Mid-low (0–1 s.d.)	238 927 (37.3%)
Mid-high (1–2 s.d.)	168 600 (26.3%)
High (2 + s.d.)	53 403 (8.3%)
Age at first registration for MD	
12–19	57 473 (9.0%)
20–29	150 434 (23.5%)
30–39	116 720 (18.2%)
40–49	110 629 (17.3%)
50–59	97 825 (15.3%)
60–69	81 340 (12.7%)
70–	26 643 (4.2%)
Treatment setting	
Inpatient	21 439 (3.3%)
Specialist care	114 521 (17.9%)
Primary care	505 104 (78.8%)
Severity	
Mild	53 059 (8.3%)
Moderate	75 356 (11.8%)
Severe	13 589 (2.1%)
Psychotic	2450 (0.4%)
No definition	496 610 (77.5%)
Prior psychiatric comorbidity	
AD	247 805 (38.7%)
ONAP	4734 (0.7%)
OCD	7602 (1.2%)
DUD	35 937 (5.6%)
AUD	45 523 (7.1%)
SA	34 353 (5.4%)
ADHD	15 807 (2.5%)

FGRS, family genetic risk scores; BD, bipolar disorder; s.d., standard deviation; MD, major depressive disorder; AD, anxiety disorder; ONAP, other nonaffective psychosis; OCD, obsessive-compulsive disorder; DUD, drug use disorder; AUD, alcohol use disorder; SA, suicide attempt; ADHD, attention deficit hyperactivity disorder.

**Table 3.** Cox regression models of BD conversion from first MD registration

	Baseline (*N* = 641 064)		After 5 years from first MD registration (*N* = 322 991)
	Univariable analysis HR (95% CI)		Multivariable analysis HR (95% CI)
BD conversion (*N*, %)	20 750 (3.24%)		4412 (1.37%)
Baseline characteristics			
Sex			
Males	**0.95 (0.93–0.98)**	**0.88 (0.84–0.91)**	**0.87 (0.79–0.95)**
Females	Reference	Reference	Reference
FGRS_BD_			
Low (−0 s.d.)	Reference	Reference	Reference
Mid-low (0–1 s.d.)	**1.74 (1.67–1.80)**	**1.48 (1.40–1.56)**	**1.45 (1.29–1.63)**
Mid-high (1–2 s.d.)	**2.42 (2.31–2.53)**	**2.03 (1.90–2.18)**	**1.75 (1.50–2.03)**
High (2 + s.d.)	**3.06 (2.82–3.32)**	**2.73 (2.43–3.08)**	**2.43 (1.86–3.17)**
FGRS_MD_			
Low (−0 s.d.)	Reference	Reference	Reference
Mid-low (0–1 s.d.)	**1.20 (1.15–1.24)**	1.05 (0.99–1.10)	0.97 (0.86–1.08)
Mid-high (1–2 s.d.)	**1.39 (1.34–1.44)**	**1.15 (1.09–1.21)**	1.02 (0.91–1.15)
High (2 + s.d.)	**1.56 (1.48–1.64)**	**1.14 (1.06–1.23)**	1.06 (0.91–1.24)
Age at first registration for MD			
12–19	**2.00 (1.90–2.10)**	**1.51 (1.40–1.63)**	**2.21 (1.88–2.59)**
20–29	**1.80 (1.72–1.87)**	**1.70 (1.60–1.80)**	**1.78 (1.56–2.02)**
30–39	**1.33 (1.27–1.39)**	**1.32 (1.23–1.40)**	**1.32 (1.15–1.51)**
40–49	Reference	Reference	Reference
50–59	**0.68 (0.64–0.71)**	**0.68 (0.63–0.73)**	**0.67 (0.57–0.80)**
60–69	**0.41 (0.38–0.44)**	**0.44 (0.40–0.49)**	**0.31 (0.24–0.40)**
70–	**0.22 (0.18–0.27)**	**0.27 (0.20–0.35)**	**0.12 (0.02–0.89)**
Treatment setting			
Inpatient	**3.53 (3.37–3.71)**	**2.64 (2.44–2.84)**	**1.45 (1.23, 1.72)**
Specialist care	**2.49 (2.41–2.56)**	**1.93 (1.83–2.02)**	**1.24 (1.11–1.39)**
Primary care	Reference	Reference	Reference
Severity			
Mild	Reference	Reference	Reference
Moderate	**1.92 (1.79–2.05)**	**1.49 (1.36–1.64)**	1.06 (0.95–1.45)
Severe	**3.01 (2.77–3.27)**	**2.16 (1.92–2.43)**	1.27 (0.96–1.68)
Psychotic	**4.34 (3.81–4.95)**	**2.58 (2.14–3.11)**	1.39 (0.88–2.21)
No definition	1.04 (0.98–1.11)	**1.36 (1.25–1.49)**	1.17 (0.95–1.45)
Prior psychiatric comorbidity			
AD	**1.25 (1.21–1.28)**	**1.14 (1.09–1.19)**	**0.89 (0.81–0.98)**
ONAP	**3.30 (3.02–3.62)**	**2.18 (1.91–2.49)**	1.42 (0.99–2.05)
OCD	**1.38 (1.23–1.55)**	0.88 (0.75–1.04)	0.92 (0.61–1.38)
DUD	**1.87 (1.78–1.96)**	**1.20 (1.12–1.29)**	0.89 (0.74–1.08)
AUD	**1.49 (1.43–1.56)**	**1.45 (1.35–1.56)**	**1.22 (1.04–1.45)**
SA	**1.88 (1.80–1.97)**	**1.28 (1.19–1.37)**	1.15 (0.99–1.34)
ADHD	**1.95 (1.81–2.10)**	0.93 (0.83–1.03)	0.86 (0.62–1.18)
0–5 years characteristics			
Number of MD registrations			
None (0)			Reference
Single (1)			**1.34 (1.18–1.54)**
2–5			**1.85 (1.65–2.07)**
6+			**2.37 (2.05–2.75)**
Prior psychiatric comorbidity			
AD			**1.41 (1.28–1.55)**
ONAP			1.02 (0.78–1.32)
OCD			0.85 (0.66–1.09)
DUD			0.99 (0.85–1.15)
AUD			**1.41 (1.22–1.62)**
SA			1.11 (0.96–1.28)
ADHD			1.17 (1.00–1.36)
Prior psychotropic medication			
Antipsychotics			**1.86 (1.66–2.09)**
Mood stabilizers			**2.92 (2.59–3.20)**
Antidepressants			**1.69 (1.44–1.98)**
Concordance statistics		0.708 (0.703;0.713)	0.791 (0.781–0.800)

BD, bipolar disorder; MD, major depressive disorder; HR, hazard ratio; CI, confidence interval; FGRS, family genetic risk scores; s.d., standard deviation; AD, anxiety disorder; ONAP, other nonaffective psychosis; OCD, obsessive-compulsive disorder; DUD, drug use disorder; AUD, alcohol use disorder; SA, suicide attempt; ADHD, attention deficit hyperactivity disorder.

Boldface HRs are significant at *p* < 0.05.

### Univariable and multivariable analyses in the total population

In the univariable analysis (Table 3), except for those individuals without a recorded severity in their ICD diagnosis, every variable considered was significantly associated with conversion to BD. This included the FGRS for BD and MD and the younger age of first MD registrations. Severe and psychotic depression [hazard ratio (HR) = 3.01 (95% confidence interval (CI) 2.77–3.27) and HR = 4.34 (95% CI 3.81–4.95), respectively], and those in inpatient or specialist outpatient cares (compared to primary care) [HR = 3.53 (95% CI 3.53–3.71) and HR = 2.49 (95% CI 2.41–2.56), respectively] were also significant risk factors for conversion. Males had a modest but significantly lower risk [HR = 0.95 (95% CI 0.93–0.98)] of conversion than females. All of the psychiatric comorbidities increased the risk of conversion.

In the multivariable analysis, every predictor variable was significantly associated with conversion with the same sign as seen in the univariable analyses, with the exception of mid-low FGRS for MD, OCD, and ADHD (Table 3). The strongest risk factors for conversion were high FGRS of BD [HR = 2.7 (95% CI 2.43–3.08)], inpatient treatment settings [HR = 2.64 (95% CI 2.44–2.84)], and psychotic depression [HR = 2.58 (95% CI 2.14–3.11)]. Those with the age of first MD registration in their 20s had the highest risk when compared with those in their 40s. When we repeated all our analyses requiring at least two independent BD registrations, the results were very similar (Appendix Table 5).

**Figure 1.**
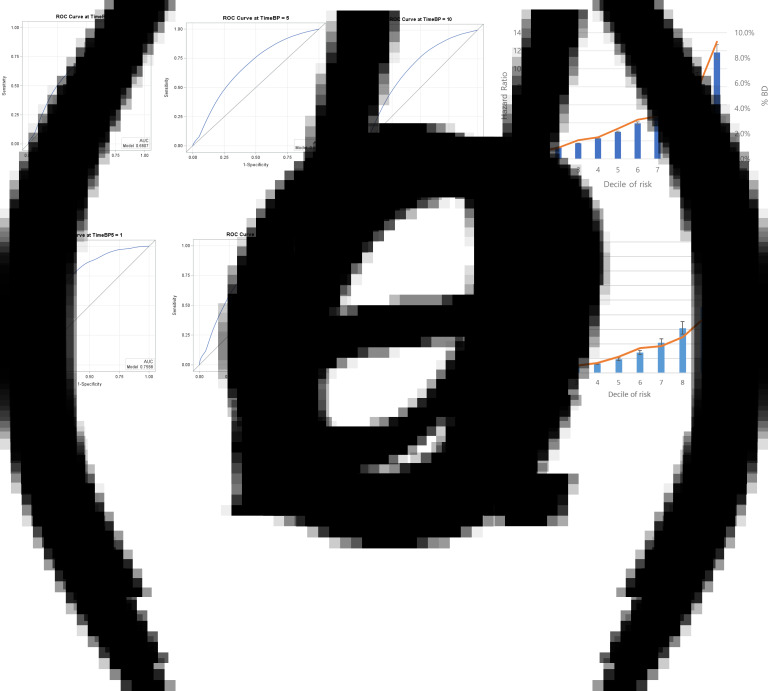
(*a*) ROC analysis of a multivariate Cox regression model for BD conversion in MD patients. ROC curves for different time points. From left: 1 year, 5 years, and 10 years after MD registration. (*b*) Multivariate Cox regression model for BD conversion in MD patients. Multivariate Cox regression model was fitted to a training sample and was then applied to a test sample. The test sample was divided into 10 risk groups based on its decile risk calculated from the multivariate Cox regression model. (*c*) ROC analysis of a multivariate Cox regression model for BD conversion in MD patients after 5 years of non-conversion. ROC curves for different time points. From left: 6 years, 10 years after MD registration. (*d*) Multivariate Cox regression model for BD conversion in MD patients after 5 years of non-conversion. A multivariate Cox regression model was fitted to a training sample and was then applied to a test sample. The test sample was divided into 10 risk groups, based on its decile risk calculated from the multivariate Cox regression model. ROC, receiver operating characteristic; BD, bipolar disorder; MD, major depressive disorder.

When we fitted multivariable Cox regression models in the training set and applied these results to the test set, the concordance statistic was 0.708 (95% CI 0.703–0.713). AUROC values ranged from 0.673 to 0.681 at different time points (Fig. 1a). When risk scores from the deciles of the linear predictor were calculated and used as predictors in a Cox regression model, the HR increased as risk scores increased. In particular, for the 9^th^ and 10^th^ decile of risk scores, the HRs were 8.98 (95% CI 8.43–9.57) and 11.81 (95% CI 11.00–12.69), respectively (Fig. 1b).

### Analysis of late converters

To examine the predictors of conversion later in the course of MD, we repeated our conversion risk analyses in the 322 911 individuals who we followed for at least five years from first MD registrations and who had up to that point no BD registrations (Table 3). This group had a 1.37% conversion rate to BD during the remaining observation period. When considering characteristics only measured at or prior to the first MD registration, the FGRS for MD and severity were no longer statistically significant. However, the FGRS for BD and treatment settings were still significant, and first MD registration during the teenage years had a higher risk when compared to the original model [HR = 2.21 (95% CI 1.88–2.59) *v.* HR = 1.51 (95% CI 1.40–1.63)]. Next, we compared comorbidities measured both at baseline and for the first five years. Prior psychiatric comorbidity for AD at baseline was a protective factor, but a risk factor for conversion during the 5-year period of non-conversion. Both AUD at baseline and during the 5-year period of non-conversion were significant risk factors. Finally, receipts of antipsychotics, mood stabilizers, and antidepressants during the 5-year period of non-conversion all increased conversion risks.

When we fitted multivariable Cox regression models in the training set and applied these results to the test set, the concordance statistic was 0.791 (95% CI 0.781–0.800). AUROC values ranged from 0.736 to 0.759 at different time points (Fig. 1c). When risk scores from the deciles of the linear predictor were calculated and used as predictors in a Cox regression model, the HR increased as risk scores increased. In particular, for the 9^th^ and 10^th^ decile of risk scores, the HRs were 22.67 (95% CI 19.33–26.59) and 33.49 (95% CI 28.00–40.06), respectively (Fig. 1d).

### Sex-stratified analysis

We first tested the significance of the interaction between the predictor variables and sex (Table 4). After adjustment for multiple comparisons, mid-low FGRS for BD, mid-high, and high FGRS for MD, earlier age of first MD registration compared with those in their 40s, treatment settings, and certain comorbidities (DUD, AUD, SA, and ADHD) were significant. When considering these variables in the multivariate analysis (Table 4), most of them had higher HRs in females than in males but were significant risk factors in both sexes. However, mid-high, and high FGRS for MD, first MD registration during the teenage years, and comorbid ADHD were significant predictors of conversion in females only. The overall risk of BD conversion was higher in females than in males (Appendix Figure 2).

**Table 4.** Stratification analysis by sex of BD conversion from first MD registration

	Univariate interaction analysis^a^		Multivariable analysis^b^	
	HR (95% CI)^a^	*p* value	Females (*N* = 402 321) HR (95% CI)	Males (*N* = 238 743) HR (95% CI)
BD Conversion (*N*, %)			13 447 (3.34%)	7303 (3.06%)
Characteristics				
FGRS_BD_				
Low (−0 s.d.)			Reference	Reference
Mid-low (0–1 s.d.)	**0.87 (0.80–0.94)**	**0.001**	**1.48 (1.42–1.55)**	**1.39 (1.30–1.48)**
Mid-high (1–2 s.d.)	0.93 (0.85–1.03)	0.164	**2.12 (2.00–2.24)**	**2.14 (1.97–2.31)**
High (2 + s.d.)	1.00 (0.95–1.19)	0.97	**2.86 (2.58–3.17)**	**2.92 (2.54–3.35)**
FGRS_MD_				
Low (−0 s.d.)			Reference	Reference
Mid-low (0–1 s.d.)	0.93 (0.86–1.00)	0.045	**1.07 (1.02–1.12)**	1.01 (0.95–1.07)
Mid-high (1–2 s.d.)	**0.84 (0.78–0.91)**	**<0.001**	**1.18 (1.12–1.24)**	1.02 (0.95–1.09)
High (2 + s.d.)	**0.85 (0.76–0.94)**	**0.002**	**1.20 (1.12–1.28)**	1.04 (0.96–1.14)
Age at first registration for MD				
12–19	**0.49 (0.44–0.55)**	**<0.001**	**1.80 (1.69–1.93)**	1.01 (0.91–1.11)
20–29	**0.62 (0.57–0.68)**	**<0.001**	**1.99 (1.88–2.10)**	**1.25 (1.17–1.34)**
30–39	**0.80 (0.73–0.87)**	**<0.001**	**1.44 (1.36–1.53)**	**1.15 (1.07–1.23)**
40–49			Reference	Reference
50–59	1.10 (0.98–1.23)	0.101	**0.65 (0.60–0.70)**	**0.71 (0.65–0.77)**
60–69	1.19 (1.03–1.38)	0.017	**0.40 (0.36–0.44)**	**0.47 (0.42–0.53)**
70–	1.24 (0.84–1.85)	0.278	**0.23 (0.17–0.30)**	**0.27 (0.20–0.36)**
Treatment setting				
Inpatient	**0.71 (0.63–0.78)**	**<0.001**	**2.82 (2.64–3.02)**	**2.26 (2.07–2.47)**
Specialist care	**0.79 (0.74–0.84)**	**<0.001**	**1.99 (1.90–2.08)**	**1.81 (1.71–1.93)**
Primary care			Reference	Reference
Severity				
Mild			Reference	Reference
Moderate	0.91 (0.79–1.05)	0.196	**1.48 (1.36–1.61)**	**1.50 (1.34–1.67)**
Severe	0.89 (0.75–1.06)	0.199	**2.15 (1.93–2.39)**	**2.12 (1.84–2.43)**
Psychotic	0.91 (0.70–1.19)	0.493	**2.73 (2.26–3.29)**	**2.65 (2.15, 3.28)**
No definition	1.05 (0.93–1.19)	0.426	**1.41 (1.31–1.52)**	**1.36 (1.23–1.51)**
Prior psychiatric comorbidity				
AD	1.01 (0.95–1.07)	0.075	**1.17 (1.13–1.22)**	**1.15 (1.09–1.21)**
ONAP	0.85 (0.69–1.01)	0.058	**2.41 (2.11–2.76)**	**1.91 (1.64–2.23)**
OCD	0.84 (0.66–1.06)	0.146	0.86 (0.75–0.99)	0.83 (0.68–1.01)
DUD	**0.73 (0.67–0.80)**	**<0.001**	**1.34 (1.24–1.44)**	**1.13 (1.05–1.22)**
AUD	**0.75 (0.68–0.82)**	**<0.001**	**1.56 (1.45–1.68)**	**1.33 (1.24–1.43)**
SA	**0.77 (0.70–0.85)**	**<0.001**	**1.33 (1.25–1.42)**	**1.16 (1.06–1.27)**
ADHD	**0.61 (0.52–0.70)**	**<0.001**	**1.11 (1.00–1.23)**	0.90 (0.80–1.02)
Concordance statistics			0.729 (0.724–0.733)	0.680 (0.673–0.686)

BD, bipolar disorder; MD, major depressive disorder; HR, hazard ratio; CI, confidence interval; FGRS, family genetic risk scores; s.d., standard deviation; AD, anxiety disorder; ONAP, other nonaffective psychosis; OCD, obsessive-compulsive disorder; DUD, drug use disorder; AUD, alcohol use disorder; SA, suicide attempt; ADHD, attention deficit hyperactivity disorder.

a HR less than unity signifies a higher risk factor for females. Boldface HRs are significant at Bonferroni corrected *p* < 0.05.

b Boldface HRs are significant at *p* < 0.05.

## Discussion

In this study, we sought to evaluate the rate and predictors of BD conversion among individuals with MD. The cumulative incidence of conversion was 5.84%, and the incidence rate of conversion decreased over time. We demonstrated that a wide range of characteristics, including demographics, familial risks, depressive features, comorbidities, and medication, were significant risk factors. There were significant differences in late converters when compared with the overall converters, and many of the analyzed factors were more predictive in females.

In our study, the rate of conversion considering follow-up duration was lower than in the meta-analysis of Kessing et al. (2017) (12.9% in 10 years) and Ratheesh et al. (2017) (22.5% in 12–18 years). It is known that register studies have lower conversion rates than recruitment-based studies, as recruitment studies sensitively and rigorously evaluate for BD conversion, in which the meta-analysis of Kessing et al. (2017) and Ratheesh et al. (2017) mostly reviewed. The rate of conversion was also lower than national representative studies from Korea (6.5% in 8 years) (Jo et al., 2022), Finland (7.4% over 15 years) (Baryshnikov et al., 2020), and Denmark (8.4% over 21 years) (Musliner & Østergaard, 2018) but similar in a study from England (5.65% over 12 years) (James, Wotton, Duffy, Hoang, & Goldacre, 2015) and another study from Denmark (6.5% over 20 years) (Kessing, 1999). These discrepancies are probably due to differences in ages and treatment settings identifying MD and BD conversions. Our study included primary care data, as it is the majority source of MD registration in Sweden, as well as a broad age range, for generalizability. These both likely contributed to our lower conversion rate as cases of MD ascertained in primary care settings are likely to be milder and less likely to convert than those MD cases ascertained in specialist clinics or as inpatients.

Interestingly, FGRS for MD and BD were both significant conversion factors for our entire sample, but only FGRS for BD was significant for later converters. Previous studies regarding family history mostly analyzed it as a dichotomous variable, based on self-reports. Moreover, previous studies rarely focused on both MD and BD risks. A study of Musliner and Østergaard (2018) based on Danish register data reviewed parents' records for a variety of psychiatric diseases, revealing only MD and BD as significant risk factors. Meanwhile, a study of Fiedorowicz et al. (2011) interviewed at least one family member and revealed that only BD and not MD family history was a significant risk factor. We were able to analyze familial genetic loading with a more improved measure based on direct information of cases in relatives for high-quality registry data, calculating phenotype risks up to the 5^th^ degree, and also considering family environmental influences (Kendler, Ohlsson, Sundquist, & Sundquist, 2021). Higher familial loadings of BD were important risk factors, even for late converters. This is line with previous genetic studies showing high heritability of BD (O'Connell & Coombes, 2021).

Our study replicated the most consistent risk factor of conversion: early age of onset. It has been replicated in the past, even though studies defined early onset heterogeneously. However, if we confine the results to register-based studies, studies from Denmark, Finland, and England have reported negative or even opposite results (Baryshnikov et al., 2020; James et al., 2015; Kessing, 1999; Musliner & Østergaard, 2018). The earlier the onset, the increased risk of conversion, but a delayed diagnosis of BD (Post et al., 2020; Suominen et al., 2007) seems to attenuate the conversion risk in those with younger onset ages. The symptom manifestation could be ambiguous, or the physician could be reluctant in diagnosing BD in these populations (Dudek, Siwek, Zielińska, Jaeschke, & Rybakowski, 2013). Due to this delay, the follow-up duration of each study could affect the results. This was reflected in our study as in overall converters, the HR was the highest for those whose first registration of MD was in their 20s, but for later converters, the HR was higher for those whose first registration of MD was in their teenage years.

Another significant risk factor was psychotic depression. Interestingly, as in our investigation, studies based on national representative registers that investigated psychotic depression all reported it as a significant risk factor (Baryshnikov et al., 2020; James et al., 2015; Kim et al., 2020b; Musliner & Østergaard, 2018). Most of the studies with negative results reported psychotic depression as a significant risk factor only in univariate analyses or as an insignificant risk factor in multivariate analyses although in the same direction as our findings (Bukh, Andersen, & Kessing, 2016; de Azevedo Cardoso et al., 2020; Holma, Melartin, Holma, & Isometsä, 2008; Takeshima et al., 2008; Wong, Dunn, Tang, Chan, & Chong, 2009; Woo et al., 2015; Xu et al., 2022). This implies that the sample size could have affected the findings from previous studies that were not based on national representative registers. Psychotic depression itself is known to have a worse prognosis and a highly recurrent course (Dubovsky, Ghosh, Serotte, & Cranwell, 2021), which is also associated with bipolarity.

One of the advantages of this study was that we were able to compare the potential effect of treatment settings on conversion risk, especially given that the majority of MD cases in our study were from primary care. Not only did our study replicate previous register studies in which the risk is higher in inpatients (Kim et al., 2020a, 2020b; Musliner & Østergaard, 2018), but also demonstrated that the risk is higher in specialist care than primary care. Even though the severity itself was controlled, the risk of conversion differed between treatment settings, which is most likely due to distinct patients' characteristics.

Although there have been more negative results in previous studies, SA was associated with BD conversion. It is well known that BD has a higher SA rate than MD (Baldessarini, Tondo, Pinna, Nuñez, & Vázquez, 2019). SAs occur much more commonly in the onset of BD – during the first depressive episode – which could explain the increased risk of conversion (Gonda et al., 2012). However, as SA was at or prior to first MD registration, this could also reflect an even earlier onset of symptoms before seeking treatment.

The risk factors previously mentioned (psychotic depression, inpatients, specialist care, and SA) can all be viewed as a more severe phenotype of depression. However, previous studies rarely analyzed depression severity itself as an independent risk factor. Our study replicated previous studies from Nordic register-based studies (Baryshnikov et al., 2020; Musliner & Østergaard, 2018), and the study of Holma et al. (2008), who analyzed 248 patients with life charts although only the objective and not the subjective scale of depressive symptoms, was significant. The study of Bukh et al. (2016), which analyzed 301 MD patients, also analyzed severity, and although the HR increased as severity increased, it was not statistically significant, probably due to the sample size.

When analyzing comorbidities, there were some different associations between overall and late converters. Although AD at or prior to first MD registration was a risk factor, it was a protective factor for late converters. The effect of AD on BD conversion differed throughout the course of MD. This might explain the discrepancies found in previous studies, including a report of comorbid AD as a protective factor in a Korean study of young adults (Kim et al., 2020b). The most significant comorbidity throughout the disease course was AUD. However, DUD was only a significant risk factor in overall converters. The study of Musliner and Østergaard (2018) reported that AUD but not DUD was a risk factor for BD conversion, and the study of Oliveira et al. (2021) and de Azevedo Cardoso et al. (2020) reported lifetime drug use but not lifetime alcohol use as a risk factor for BD conversion. Our study not only shows that AUD and DUD effects should be analyzed separately but also that their effect on BD conversion differs throughout the course of MD. ONAP was a significant risk factor for overall BD conversion, replicating the results from the study of Musliner and Østergaard (2018), which is also in line with psychotic symptoms predicting BD. However, OCD and ADHD were only significant in the univariate analysis, which implies that these effects were possible confounded with those of other risk factors.

Antipsychotics, mood stabilizers, and antidepressants all increased the risk of conversion. Antidepressant monotherapy is known to increase (hypo)manic episodes (Baldessarini et al., 2013); therefore, its significance was expected. Interestingly, the HR for mood stabilizers and antipsychotics was even stronger. As reported in the study of Kim et al. (2020a), the use of mood stabilizers and antipsychotics increases right before BD diagnosis, which might reflect the prescription of these medications in treatment-resistant depression. Physicians could also be aware of a potential bipolarity and prescribe these medications before providing a bipolar diagnosis. Nevertheless, the stronger association of mood stabilizers/antipsychotics prescription should be noticed, and physicians should be aware of the elevated risk of conversion when using these medications.

A higher number of registrations, which is a good proxy for the number of illness episodes, was associated with a higher BD conversion risk. Although previous studies have had mixed results, nationwide register-based studies that compared single *v.* recurrent MD based on ICD codes reported a higher risk for recurrent MD (Baryshnikov et al., 2020; Kim et al., 2020b; Musliner & Østergaard, 2018). When compared to MD, BD is known to be more episodic and to have a recurrent/cyclic nature (Hirschfeld, 2014).

The conversion rate itself was slightly higher in females, which has had mixed results in previous studies. Interestingly, there was no risk factor that was stronger in males. The course of BD is known to have more depressive phases in females, and females are prone to have a higher risk of major depressive episodes in a lifetime, which sometimes is related to hormonal changes, such as menarche, post-partum, menopause, etc. (Swaab & Bao, 2020). Early onset could be a stronger risk factor for BD in females. Moreover, low FGRS of MD/BD, primary care settings, and fewer comorbidities might reflect a milder state of MD with lower risks of bipolarity, in which females might be more representative.

Overall, the risk values indicated that the accumulation of risk factors resulted in a 10-fold difference in conversion. For late-converters, the conversion risk was lower, but the accumulation risk differences between low and high decile groups in the linear predictor were even larger. This could be explained as we added other characteristics as especially each medication group increased the risk of conversion. The concordance index of our models showed good predictability (concordance index ≥0.7) with the exception of male converters (Zhou et al., 2019).

The study should be cautiously interpreted due to the following limitations. First, the diagnosis validity depends on the quality of the Swedish registries used in this study. Additionally, the diagnosis was clinically based, and the utilization rate of structural diagnostic interviews is not known. However, the diagnosis of BD based on these registers is well supported (Sellgren, Landén, Lichtenstein, Hultman, & Långström, 2011). The validity of MD is supported by their prevalence, sex ratio, sibling/twin correlations, and associations with known psychosocial risk factors (Kendler, Ohlsson, Lichtenstein, Sundquist, & Sundquist, 2018; Sundquist, Ohlsson, Sundquist, & Kendler, 2017). Nevertheless, mildly affected patients in the community could be underrepresented. Furthermore, the primary care data were not available with complete nationwide coverage, and coverage was less complete in the earlier years. Thus, the conversion rate could have been underestimated. Second, there are other known risk factors that could not be retrieved from registers. Mixed/subthreshold hypomanic symptoms (Fiedorowicz et al., 2011; Nakamura, Iga, Matsumoto, & Ohmori, 2015; Takeshima et al., 2008), diurnal mood (Gan et al., 2011; Pfennig et al., 2016), treatment-resistance depression (Bukh et al., 2016; Dudek et al., 2013; Woo et al., 2015), etc., were not analyzed, in which case-control cohort studies could assess in a more comprehensive way. Third, the duration of follow-up might not have been sufficient enough to catch all BD conversions. However, most studies suggest that the majority converts within a short period, and the rate of conversion decreases over time (Kessing et al., 2017; Ratheesh et al., 2017). Nevertheless, studies that were conducted with a longer duration have shown that conversion may continue even after a long time (Angst, Sellaro, Stassen, & Gamma, 2005; Dudek et al., 2013; Tse, Fok, Yim, Leung, & Leung, 2021). Fourth, specific subtypes of bipolar disorder (bipolar I *v.* II disorder) could not be analyzed separately due to the limitation of ICD diagnostic codes. Finally, this study is limited to the Swedish population and may not easily be generalized to other countries.

## Conclusions

After the dichotomy of MD and BD was established a long time ago, efforts to differentiate these diseases and to investigate the conversion of MD to BD have been an ongoing task. Our study, which included a substantial number of MD patients and utilized FGRS, demonstrated that the accumulation of risk factors implied a substantially increased risk of BD conversion. Clinically, those with a potentially higher risk of conversion should be monitored closely for the onset of manic episodes.

## Supporting information

Rhee et al. supplementary material Rhee et al. supplementary material

## Supplementary material

The supplementary material for this article can be found at https://doi.org/10.1017/S0033291723001848.
