# Supplementary material for: Predictors of diagnostic conversion from major depression to bipolar disorder: a Swedish national longitudinal study
Source: Psychol Med. 2023 Jul 10;53(16):7805–16. doi: 10.1017/S0033291723001848 (PMC10755232; doi:10.1017/S0033291723001848)
Supplement: Rhee et al. supplementary material [file S0033291723001848sup001.docx]

**Appendix**

**Table 1 - Description of Registers**

*National Patient Register*

In the 1960s the National Board of Health and Welfare started to collect information regarding in-patients at public hospitals - the National Patient Register (NPR). Initially, the NPR contained information about all patients treated in psychiatric care and approximately 16 percent of patients in somatic care. The register at that time covered six of the 26 county councils in Sweden. In 1984, the Ministry of Health and Welfare together with the Federation of County Councils decided upon mandatory participation for all county councils. From 1987 onwards, the NPR includes all in-patient care in Sweden. Since 2001, the register also covers outpatient doctor visits including day surgery and psychiatric care from both private and public caregivers. For more information, see *https://www.socialstyrelsen.se/en/statistics-and-data/registers/register-information/the-national-patient-register/*

*Primary care data*

We also used information from our new primary care dataset, a research dataset including individual-level information on clinical diagnoses from primary health care centers from the following 20 of the 21 Swedish counties: Stockholm (2003-2018), Uppsala (2007-2018), Södermanland (2000-2018), Östergötland (2003-2018), Jönköping (2009-2018), Kronoberg (2006-2018), Kalmar (2008-2018), Gotland (2011-2018), Blekinge (2009-2018), Skåne (2000-2018), Halland (2009-2018), Västra Götaland (2000-2018), Värmland (2005-2018), Örebro (2007-2018), Västmanland (2015-2018), Dalarna (2005-2018), Gävleborg (2010-2018), Västernorrland (2011-2018), Jämtland (-), Västerbotten (2000-2018), Norrbotten (2007-2018. At the end of the follow-up period, the registers cover almost 100% of the population.

| Year | % of population in Counties with Primary Care registration |
| --- | --- |
| 2006 | 69% |
| 2007 | 79% |
| 2008 | 81% |
| 2009 | 90% |
| 2010 | 93% |
| 2011 | 96% |
| 2012 | 96% |
| 2013 | 96% |
| 2014 | 96% |
| 2015 | 99% |
| 2016 | 99% |
| 2017 | 99% |
| 2018 | 99% |

*The National Prescribed Drug Register*

[*https://www.socialstyrelsen.se/en/statistics-and-data/registers/national-prescribed-drug-register*](https://www.socialstyrelsen.se/en/statistics-and-data/registers/national-prescribed-drug-register)

The National Prescribed Drug Register provides the basis for the official statistics about prescribed drugs in Sweden. The National Prescribed Drug Register with personal identity numbers was established in July 2005 and contains all prescribed drugs dispensed at pharmacies. About 67 percent (6.8 million) of the population were prescribed a pharmaceutical at least once in 2019. Each row in the register corresponds to one dispensation at a pharmacy. The number of rows in the register amounts to more than 100 million per year. This level has remained almost unchanged for several years. The register contains information about the following: patient (gender, age, place of residence); product (for example ATC code, drug name, strength, pack size, included/not included in the pharmaceutical benefits scheme); the prescription (for example prescribed quantity/number of packages, strength, date of prescription, date of purchase and type of ordination; the costs (total cost, patient cost, region’s cost and additional patient cost); the prescriber (work title, kind of care according to the work-place, education code and specialist education code).

**Table 2 - Definition of Variables**

|  | Registers Used | Definition |
| --- | --- | --- |
| Major Depression (MD) | The Swedish Hospital Discharge Register (coverage 1973-2018); Outpatient Care Register (national coverage 2001-2018); primary care data (Partial coverage from 1999-2018) | ICD-8: 296.2, 298.0, 300.4; ICD-9: 296.2, 296.4, 298.0, 300.4; ICD-10: F32, F33. |
| Site of MD registration | The Swedish Hospital Discharge Register (coverage 1973-2018); Outpatient Care Register (national coverage 2001-2018); primary care data (Partial coverage from 1999-2018) | The variable is a three category variable (registration in Inpatient case, Specialist Care, or Primary Care |
| Psychotic MD | The Swedish Hospital Discharge Register (coverage 1973-2018); Outpatient Care Register (national coverage 2001-2018); primary care data (Partial coverage from 1999-2018) | ICD-10: F32.3, F33.3 |
| Mild MD | The Swedish Hospital Discharge Register (coverage 1973-2018); Outpatient Care Register (national coverage 2001-2018); primary care data (Partial coverage from 1999-2018) | ICD-10: F32.0, F33.0 |
| Moderate MD | The Swedish Hospital Discharge Register (coverage 1973-2018); Outpatient Care Register (national coverage 2001-2018); primary care data (Partial coverage from 1999-2018) | ICD-10: F32.1, F33.1 |
| Severe MD | The Swedish Hospital Discharge Register (coverage 1973-2018); Outpatient Care Register (national coverage 2001-2018); primary care data (Partial coverage from 1999-2018) | ICD-10: F32.2, F33.2 |
| Other MD | The Swedish Hospital Discharge Register (coverage 1973-2018); Outpatient Care Register (national coverage 2001-2018); primary care data (Partly coverage from 1999-2018) | All registrations in the MD definition not defined as Psychotic, Mild, Moderate, Severe |
| Bipolar Disorder (BD) | The Swedish Hospital Discharge Register (coverage 1973-2018); Outpatient Care Register (national coverage 2001-2018); primary care data (Partial coverage from 1999-2018) | ICD-8: 296.1, 296.3, 296.8, 296.9, 298.1; ICD-9: 296A, 296C, 296D, 296E, 296W, 298B; ICD-10: F30, F31 |
| Anxiety Disorder (AD) | The Swedish Hospital Discharge Register (coverage 1973-2018); Outpatient Care Register (national coverage 2001-2018); primary care data (Partial coverage from 1999-2018) | ICD-8: 300.0, 300.2 ; ICD-9: 300A, 300C; ICD-10: F40, F41 |
| Obsessive-Compulsive Disorder (OCD) | The Swedish Hospital Discharge Register (coverage 1973-2018); Outpatient Care Register (national coverage 2001-2018); primary care data (Partial coverage from 1999-2018) | ICD-9: 300D; ICD-10: F42 |
| Other Non-affective psychosis (ONAP) | The Swedish Hospital Discharge Register (coverage 1973-2018); Outpatient Care Register (national coverage 2001-2018); primary care data (Partial coverage from 1999-2018) | ICD-8: 297, 298.3, 298.9, 295.4, 295.7; ICD-9: 298E, 298W, 298X, 295E, 295H, 295W; ICD-10: F22, F23, F24, F25, F26, F27, F28, F29, F208. |
| Drug Use Disorder (DUD) | The Swedish Hospital Discharge Register (coverage 1973-2018); Outpatient Care Register (national coverage 2001-2018); primary care data (Partial coverage from 1999-2018); The National Prescribed Drug Register (2005-2018); the Swedish Mortality Register, and the Swedish Criminal Register (1973-2018) and the Swedish Suspicion Register (1998-2018) | DUD was identified in the Swedish medical and mortality registries by ICD codes (ICD8: Drug dependence (304); ICD9: Drug psychoses (292) and Drug dependence (304); ICD10: Mental and behavioral disorders due to psychoactive substance use (F10-F19), except those due to alcohol (F10) or tobacco (F17)); in the Suspicion Register by codes 3070, 5010, 5011, and 5012, that reflect crimes related to DA; and in the Crime Register by references to laws covering narcotics (law 1968:64, paragraph 1, point 6) and drug-related driving offenses (law 1951:649, paragraph 4, subsection 2 and paragraph 4A, subsection 2). DA was identified in individuals (excluding those suffering from cancer) in the Prescribed Drug Register who had retrieved (on average) more than four defined daily doses a day for 12 months from either of Hypnotics and Sedatives (Anatomical Therapeutic Chemical (ATC) Classification System N05C and N05BA) or Opioids (ATC: N02A). |
| Alcohol Use Disorder (AUD) | The Swedish Hospital Discharge Register (coverage 1973-2018); Outpatient Care Register (national coverage 2001-2018); primary care data (partial coverage from 1999-2018); the Swedish Drug Register (2005-2018); the Swedish Mortality Register, and the Swedish Criminal Register (1973-2018) and the Swedish Suspicion Register (1998-2018) | Alcohol Use Disorder (AUD) was identified in the Swedish medical and mortality registries by ICD codes: ICD9: V79B, 305A, 357F, 571A-D, 425F, 535D, 291, 303, 980; ICD 10: E244, G312, G621, G721, I426, K292, K70, K852, K860, O354, T51, F10); in the Crime Register by codes 3005, 3201, which reflect crimes related to alcohol abuse; in the Suspicion Register by codes 0004, 0005 (Only those individuals with at least two alcohol-related crimes or suspicion of crimes from both Crime Register and Suspicion Register were included); in the Prescribed Drug Register by the drugs disulfiram (Anatomical Therapeutic Chemical (ATC) Classification System N07BB01), acamprosate (N07BB03), and naltrexone (N07BB04). |
| Suicide Attempt (SA) | The Swedish Hospital Discharge Register (coverage 1973-2018); Outpatient Care Register (national coverage 2001-2018); primary care data (Partial coverage from 1999-2018) | IC10: X60-X84 and Y10-Y34  ICD9: E950-E959 and E980-E989  (Registrations that were followed by a suicide death within 30 days were not counted). |
| Attention deficit hyperactivity disorder (ADHD) | The Swedish Hospital Discharge Register (coverage 1973-2018); Outpatient Care Register (national coverage 2001-2018); primary care data (Partial coverage from 1999-2018) | ICD-9: 314; ICD-10: F90 |
| Schizophrenia (SZ) | The Swedish Hospital Discharge Register (coverage 1973-2018); Outpatient Care Register (national coverage 2001-2018); primary care data (Partial coverage from 1999-2018) | ICD-8: 295.1, 295.2, 2953, 295.9, 295.6; ICD-9: 295B, 295C, 295D, 295G, 295X; ICD-10: F200, F201, F202, F203, F205, F209. |
| Antipsychotics | The National Prescribed Drug Register (2005-2018) | ATC-code N05A excluding N05AN01. |
| Antidepressants | The National Prescribed Drug Register (2005-2018) | ATC-code N06A |
| Mood stabilizers | The National Prescribed Drug Register (2005-2018) | ATC-codes: N03AG01, N03AX09, N03AF01, N03AF02, N05AN01 |

**Table 3 - Calculation of the Family Genetic Risk Score (FGRS)**

|  |
| --- |
| The dataset for the calculations includes:  Column 1 = Identification number of the proband (Born 1940-2000)  Column 2 = Identification number of the relative (1st to 5th degree relatives)  Column 3 = Proportion of shared additive genetic effects (0.03125 to 0.50) with the proband  Column 4 = Year of Birth of relative  Column 5 = Sex of relative  Column 6 = Age at registration for trait  Column 7 = Age at end of follow-up (2018-12-31 or age at death, or age at emigration whichever came first) |
| **Step 1:** Using all unique relatives with a registration for the disorder, we non-parametrically estimated the distribution of *Age at first registration*. The empirical distribution is used to obtain weights for relatives without a registration for the disorder, in order to account for the proportion of the time-at-risk period they had completed at the end of follow-up. For example, for relatives at age x at end of follow-up, the weight corresponds to the proportion of relatives registered for the trait that had been registration at age x. For relatives born prior to 1958 we subtracted age at the end of follow-up with the following formula: 1958 - Year of birth of relative. This modification was done in order to control for registration effects (i.e, most registers in Sweden started in 1973 suggesting that relatives from early birth cohorts do not have the possibility to be registered at younger ages). Note that all relatives with the disorder are weighted one. |
| **Step 2:** Transform the binary variable (trait yes/no) into a z-score based on the threshold for each trait. The underlying liability of the individual is not assessable. Instead we estimated the mean of the underlying liability to obtain sex and birth decade specific Z-scores for relatives with the trait registration and relatives without the trait. We generate n random numbers from a N(0, 1) distribution and estimate the mean for relatives registered with the disorder (i.e., mean of the observations above the threshold) and for relatives without a registration (i.e., mean of all observation below the threshold). The thresholds are calculated for each decade of birth and sex. |
| **Step 3**: Correct for cohabitation effects. To estimate the cohabitation effect (i.e. “shared environment”), we created a database with all individuals in the Swedish population born in Sweden 1955-1990. We also included the number of years, during ages 0-15, that individuals resided in the same household as their biological father. We thereby were able to define two kinds of families i) “not-lived-with” father families (offspring never resided for more than 1 year in the same household or in the same community as their biological father); ii) “lived-with” father (offspring resided a minimum of 13 years in the same household as their biological father. We performed a logistic regression model with the binary trait in offspring as outcome and the binary trait in father, type of father, and their interaction as predictors. We used the interaction term as the difference of effect between genes only and genes + environment. The same approach was performed for half-siblings where we compared those who were reared together versus those reared apart. The following interaction terms were used in the calculations for each of our disorders:   \|  \|  \| Parent/Children \| Siblings \| \| --- \| --- \| --- \| --- \| \| MD \|  \| .90 \| .89 \| \| BD \|  \| .67 \| .77 \| |
| **Step 4:** Calculate the product for each relative using the four components:   1. Z-score (reflecting sex and year of birth adjusted rates) 2. Weight (reflecting the proportion of risk period they had completed) 3. Cohabitation effects 4. Proportion of shared genetic effects (0.03125 – 0.5) with the proband |
| **Step 5:** Average the product calculated in step 4 across all relatives to a proband |
| **Step 6**: Correct for the number of relatives. We multiplied the results from step 5 with a shrinkage factor. Shrinkage factor (SF): B/(B+A/C). It produces more shrinkage if B and C are small and A is large.   1. the variance of the z-score of the disorder across all relatives, 2. the variance in the mean z-score across all probands, 3. the weighted number of relatives for each proband (sum of Column 3 across each proband). |
| **Step 7:** Correct for difference by year of birth and county differences. There are 21 counties in Sweden. For each proband we used the county they had resided in during the maximum number of years (measured from 1969 and onwards) We standardized the risk score by year of birth and county of the proband into a z-score with mean 0 and SD 1. |

K-means clustering for partition the FGRSs

We used a K-means clustering to partition the FGRSs into k clusters in which each individual belongs to the cluster with the nearest mean value. K-means clustering minimizes the within-cluster variances. We selected the number of clusters based on the elbow method (the inclusion of more clusters does not lead to a significant decrease in within-cluster sum of squares). For both MD and BD this means that we will use 4 clusters. From the standardized score, the thresholds for FGRS_MD_ were -0.427; 0.526; 1.871 and for FGRS_BD_ 0.399; 2.100; 4.911.

**Table 4 - Mean number of affected 1st - 4th degree relatives in the 4 different groups of the FGRS_MD_ and FGRS_BD_**

| FGRS | Group | 1^st^ degree | 2^nd^ degree | 3^rd^ degree | 4^th^ degree |
| --- | --- | --- | --- | --- | --- |
| MD | Low | 1.00 | 0.31 | 0.62 | 0.62 |
|  | Mid-Low | 1.11 | 1.13 | 1.37 | 1.37 |
|  | Mid-High | 1.59 | 1.65 | 1.64 | 1.12 |
|  | High | 2.37 | 1.77 | 1.12 | 0.50 |
| BD | Low | 0.01 | 0.02 | 0.04 | 0.06 |
|  | Mid-Low | 0.19 | 0.51 | 0.50 | 0.21 |
|  | Mid-High | 0.73 | 0.54 | 0.25 | 0.10 |
|  | High | 1.20 | 0.56 | 0.17 | 0.04 |

Abbreviations: BD = bipolar disorder, MD = major depressive disorder, FGRS = family genetic risk scores

**Table 5 - Cox regression models of BD conversion from first MD registration, conversion based on at least two BD registrations**

| Characteristics | Original model  HR (95% CI) | BD conversion based on at least two BD registrations HR (95% CI) |
| --- | --- | --- |
| BD conversion (N, %) | 20,750 (3.24%) | 14,570 (2.27%) |
| Sex |  |  |
| Males | **0.88 (0.84; 0.91)** | **0.84 (0.81; 0.87)** |
| Females | Reference | Reference |
| FGRS_BD_ |  |  |
| Low (-0 SD) | Reference | Reference |
| Mid – Low (0-1 SD) | **1.48 (1.40; 1.56)** | **1.50 (1.43; 1.57)** |
| Mid - High (1-2 SD) | **2.03 (1.90; 2.18)** | **2.31 (2.19; 2.44)** |
| High (2+ SD) | **2.73 (2.43; 3.08)** | **3.12 (2.84; 3.44)** |
| FGRS_MD_ |  |  |
| Low (-0 SD) | Reference | Reference |
| Mid – Low (0-1 SD) | 1.05 (0.99; 1.10) | **1.06 (1.02; 1.11)** |
| Mid - High (1-2 SD) | **1.15 (1.09; 1.21)** | **1.12 (1.07; 1.18)** |
| High (2+ SD) | **1.14 (1.06; 1.23)** | **1.14 (1.07; 1.21)** |
| Age at first registration for MD |  |  |
| 12-19 | **1.51 (1.40; 1.63)** | **1.44 (1.35; 1.54)** |
| 20-29 | **1.70 (1.60; 1.80)** | **1.66 (1.58; 1.75)** |
| 30-39 | **1.32 (1.23; 1.40)** | **1.33 (1.26; 1.40)** |
| 40-49 | Reference | Reference |
| 50-59 | **0.68 (0.63; 0.73)** | **0.63 (0.59; 0.67)** |
| 60-69 | **0.44 (0.40; 0.49)** | **0.38 (0.35; 0.42)** |
| 70- | **0.27 (0.20; 0.35)** | **0.16 (0.12; 0.22)** |
| Treatment setting |  |  |
| Inpatient | **2.64 (2.44; 2.84)** | **2.58 (2.42; 2.75)** |
| Specialist Care | **1.93 (1.83; 2.02)** | **1.92 (1.84; 2.01)** |
| Primary Care | Reference | Reference |
| Severity |  |  |
| Mild | Reference | Reference |
| Moderate | **1.49 (1.36; 1.64)** | **1.52 (1.40; 1.65)** |
| Severe | **2.16 (1.92; 2.43)** | **2.09 (1.89; 2.31)** |
| Psychotic | **2.58 (2.14; 3.11)** | **2.79 (2.38; 3.27)** |
| No definition | **1.36 (1.25; 1.49)** | **1.38 (1.28; 1.48)** |
| Prior psychiatric comorbidity |  |  |
| AD | **1.14 (1.09; 1.19)** | **1.16 (1.12; 1.20)** |
| ONAP | **2.18 (1.91; 2.49)** | **2.18 (1.94; 2.45)** |
| OCD | 0.88 (0.75; 1.04) | 0.81 (0.71; 0.93) |
| DUD | **1.20 (1.12; 1.29)** | **1.10 (1.03; 1.18)** |
| AUD | **1.45 (1.35; 1.56)** | **1.46 (1.37; 1.55)** |
| SA | **1.28 (1.19; 1.37)** | **1.31 (1.23; 1.39)** |
| ADHD | 0.93 (0.83; 1.03) | 1.05 (0.96; 1.15) |
|  |  |  |
| Concordance | 0.708 (0.703; 0.713) | 0.716 (0.712; 0.721) |

| Abbreviations : BD = bipolar disorder, MD = major depressive disorder, HR = hazard ratio, CI = confidence interval, FGRS = family genetic risk scores, SD = standard deviation, AD = anxiety disorder, ONAP = other nonaffective psychosis, OCD = obsessive-compulsive disorder, DUD = drug use disorder, AUD = alcohol use disorder, SA = suicide attempt, ADHD = attention deficit hyperactivity disorder |
| --- |

Boldface HRs are significant at p < 0.05

**Figure 1 - Inverse Kaplan-Meier Curves of BD conversion**


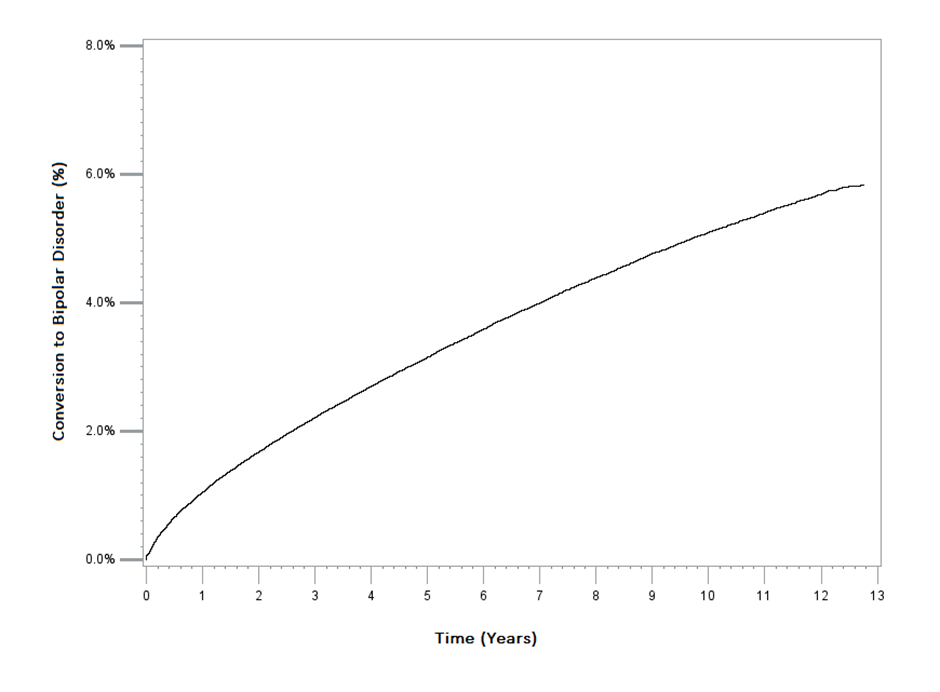


**Figure 2 - Inverse Kaplan-Meier curves of BD conversion for males and females separately**


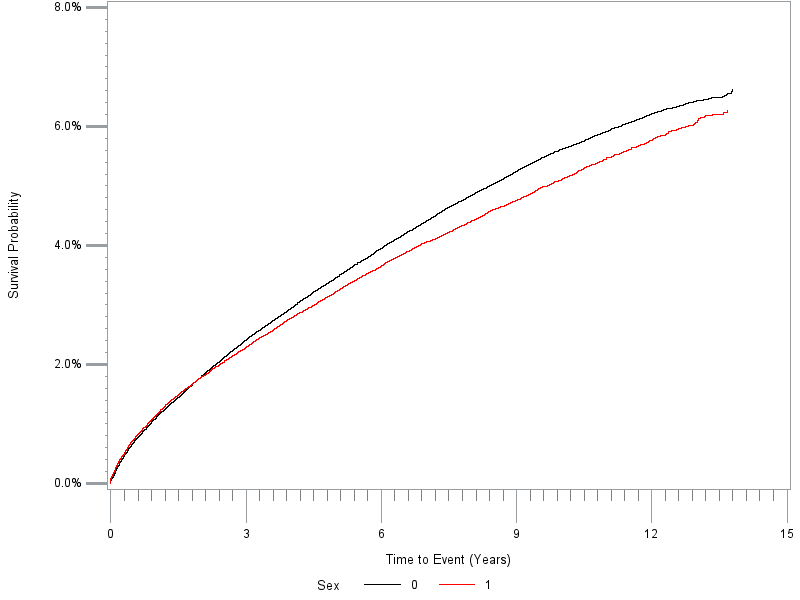


Males = Red, Females = Black
